# Pseudoaneurysm of the Anterior Tibial Artery following Tibio-Talar-Calcaneum Fusion with a Retrograde Nail: A Rare Case and Literature Review

**DOI:** 10.1155/2013/569586

**Published:** 2013-07-25

**Authors:** Simon Craxford, Saravana V. Karuppiah, Stephen Milner

**Affiliations:** Department of Orthopaedics Royal Derby Hospital, Uttoxeter Road, Derby DE22 3NE, UK

## Abstract

This study reports the case of an 87-year-old woman who presented with a nonresolving haematoma 13 weeks following tibiotalar arthrodesis surgery on her right ankle using a retrograde nail. This was revealed by angiography to be a pseudoaneurysm of the anterior tibial artery. The patient subsequently underwent endovascular stenting of the pseudoaneurysm and has had a successful recovery. This case highlights the need for awareness of both the normal arterial supply to the leg and ankle as well as the potential for anatomical variations. Arterial variation may be as high as 6.7% based on published findings from cadaveric studies. As pseudoaneurysm is a rare complication, a high index of suspicion is needed in order to avoid a missed or delayed diagnosis. We urge surgeons to keep in mind the potential for pseudoaneurysm when a patient presents with a nonresolving haematoma and arrange appropriate further investigations as needed.

## 1. Introduction

A pseudoaneurysm is a rare complication following ankle surgery. Cases of pseudoaneurysm following ankle arthroscopy [1], application of Ilizarov external fixator [2], fracture [3], and sprain of the ankle [4] have all been previously reported. We present a case of pseudoaneurysm of the anterior tibial artery after tibio-talar-calcaneal arthrodesis using a retrograde nail. The patient was treated with endovascular stenting and had a full recovery. We also review the potential variations in the course of the anterior tibial artery.

## 2. Case Presentation

**Figure 1:**
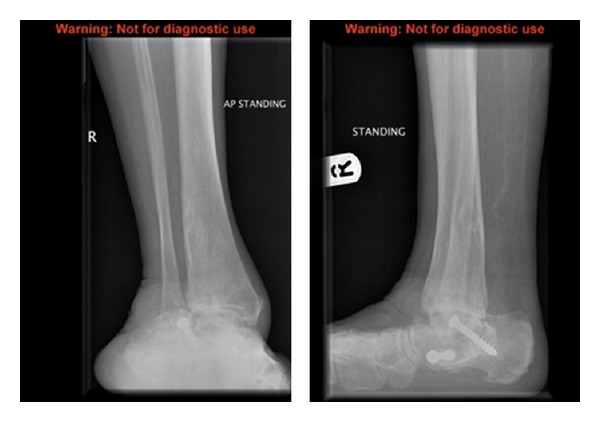
Preoperative radiograph demonstrating previous arthrodesis screws.

An 87-year-old woman in good previous health with known rheumatoid arthritis presented to the orthopaedic outpatients department. She complained of long standing pain from her right ankle. She had previously successfully undergone an elective Right Subtalar Arthrodesis eight years ago, (as shown in Figure 1) however, she was now symptomatic from tibiotalar ankle arthritis.

A tibio-talar-calcaneal arthrodesis was performed with a retrograde nail through the heel and the old arthrodesis screws were removed. There were no noted intraoperative or immediate postoperative complications. As per standard departmental operating procedure, she was immobilised in plaster for 6 weeks. At followup six weeks after surgery, a haematoma was noted at the site of the proximal locking screw, but her wound was otherwise healing well. A followup at 13 weeks showed complete union of the arthrodesis.

She, however, complained of a throbbing sensation on the leg. On examination pulsatile swelling measuring 6 cm by 6 cm was seen over the lower anterolateral region of her shin. There was an audible bruit on auscultation.

**Figure 2:**
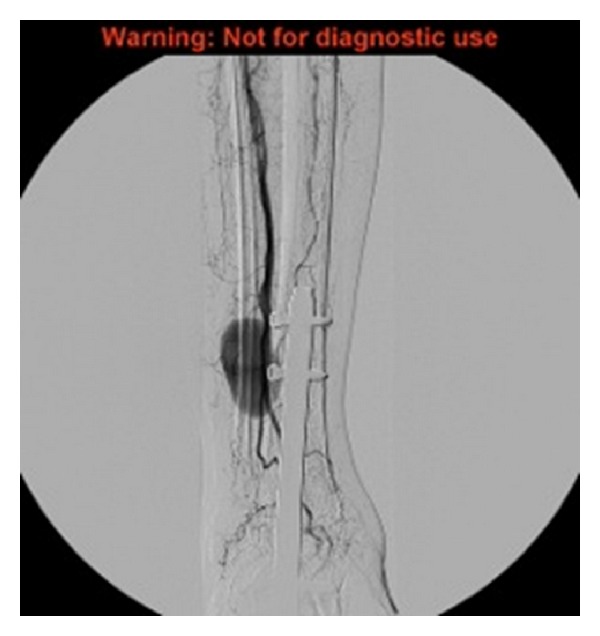
Angiography demonstrating a pseudoaneurysm of the anterior tibial artery.

**Figure 3:**
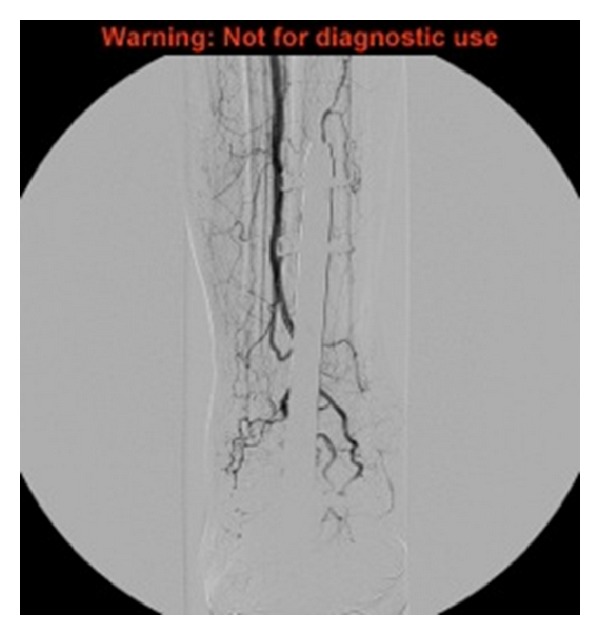
Following endovascular stenting, the artery is patent with no filling of the pseudoaneurysm.

Angiogram demonstrates a large pseudoaneurysm arising from the distal anterior tibial artery, as shown in Figure 2. The peroneal artery was patent. The posterior tibial artery is occluded distally although this reforms at the ankle joint via collaterals from the peroneal artery. She subsequently underwent stenting with a 3.5 mm Jomed covered stent on a 0.014 system, successfully deployed across the origin of the pseudoaneurysm. Postprocedure imaging demonstrated a patent anterior tibial artery with no filling of the pseudoaneurysm, as shown in Figure 3. At the last clinical followup she was asymptomatic with good perfusion to the foot.

## 3. Discussion

Pseudoaneurysm following ankle surgery is a rare complication. It may present as a pulsatile and painful swelling after surgery or may be confused with a haematoma, leading to a delay in diagnosis [5]. Previous cases of pseudoaneurysm have been reported following intramedullary nailing for tibial [6] and femoral fractures [7]. After an extensive search of the available literature, we believe that our case may be the first reported pseudoaneurysm following a retrograde nail used in ankle fusion.

The usual anatomy of the foot and ankle is well documented; however, it is important to keep in mind the potential variations in blood supply to this region. The anterior tibial artery commences at the bifurcation of the popliteal artery. It descends in the leg on the anterior surface of the interosseus membrane. It is most superficial at the foot-ankle joint, where it becomes the dorsalis pedis pulse in the foot. Several cadaveric studies have reported variations in the course of the tibial artery and its branches. A study of 150 cadavers by Vazquez et al. in 2006 found that in 2% of cadavers the anterior tibial artery had a more lateral course, running in front of the lateral malleolus. In the same study, it was observed that in 1.3% of cadavers, the perforating branch of the peroneal artery assumed the expected course of the dorsalis pedis artery, and in 1% the anterior tibial artery gave a lateral branch that replaced the perforating branch of the peroneal artery to supply the lateral aspect of the ankle. This gave a total incidence in the variation of the anterior tibial artery of around 5% [8]. A study by Yamada et al. found that dorsalis pedis was absent in 6.7% of cadavers [9].

The management of pseudoaneurysm is varied. While some may spontaneously thrombose that [10] the usual treatment consists of either surgical or radiological intervention [11]. Endovascular therapies include coil embolisation [12], thrombin injection [13], or, as in our case, endovascular stent insertion [14]. Prompt management of a proven pseudoaneurysm is needed due to the progressive risk of enlargement and eventual rupture [6].

In conclusion, a pseudoaneurysm is a rare complication of tibio-talar-calcaneal arthrodesis using a retrograde nail. It may present early or late in the postoperative period and needs to be part of a differential diagnosis for a nonresolving haematoma. Due to the rarity of this complication, it may be easily missed or diagnosed late, as in our case. Surgeons who carry out arthrodesis of the ankle using a retrograde nail should be aware of variations in arterial supply to this region as the patient may still be at risk of arterial injury despite “safe” placement of screws.
